# Regulation of Anthrax Toxin-Specific Antibody Titers by Natural Killer T Cell-Derived IL-4 and IFNγ

**DOI:** 10.1371/journal.pone.0023817

**Published:** 2011-08-17

**Authors:** T. Scott Devera, Sunil K. Joshi, Lindsay M. Aye, Gillian A. Lang, Jimmy D. Ballard, Mark L. Lang

**Affiliations:** Department of Microbiology and Immunology, University of Oklahoma Health Sciences Center, Oklahoma City, Oklahoma, United States of America; Albany Medical College, United States of America

## Abstract

Activation of Natural Killer-like T cells (NKT) with the CD1d ligand α-GC leads to enhanced production of anthrax toxin protective Ag (PA)-neutralizing Abs, yet the underlying mechanism for this adjuvant effect is not known. In the current study we examined the role of Th1 and Th2 type responses in NKT-mediated enhancement of antibody responses to PA. First, the contribution of IL-4 and IFNγ to the production of PA-specific toxin-neutralizing Abs was examined. By immunizing C57Bl/6 controls IL-4^−/−^ mice and IFNγ^−/−^ mice and performing passive serum transfer experiments, it was observed that sera containing PA-specific IgG1, IgG2b and IgG2c neutralized toxin in vitro and conferred protection in vivo. Sera containing IgG2b and IgG2c neutralized toxin in vitro but were not sufficient for protection in vivo. Sera containing IgG1 and IgG2b neutralized toxin in vitro and conferred protection in vivo. IgG1 therefore emerged as a good correlate of protection. Next, C57Bl/6 mice were immunized with PA alone or PA plus a Th2-skewing α-GC derivative known as OCH. Neutralizing PA-specific IgG1 responses were modestly enhanced by OCH in C57Bl/6 mice. Conversely, IgG2b and IgG2c were considerably enhanced in PA/OCH-immunized IL-4^−/−^ mice but did not confer protection. Finally, bone marrow chimeras were generated such that NKT cells were unable to express IL-4 or IFNγ. NKT-derived IL-4 was required for OCH-enhanced primary IgG1 responses but not recall responses. NKT-derived IL-4 and IFNγ also influenced primary and recall IgG2b and IgG2c titers. These data suggest targeted skewing of the Th2 response by α-GC derivatives can be exploited to optimize anthrax vaccination.

## Introduction

The protective Ag (PA) protein secreted by *Bacillus anthracis* is an 83 kDa protein that forms heptameric pores on the surface of target cells expressing anthrax toxin receptors (capillary morphogenesis protein-2, CMG-2 and tumor endothelial marker-8,TEM-8) [1], [2]. PA heptamers interact with lethal factor (LF) and edema factor (EF) to form lethal toxin (LT) and edema toxin (ET), which together are referred to as anthrax toxin [2]. The PA heptamer facilitates entry of EF and LF into the target cell. Following cell entry, EF generates supra-physiological levels of cAMP via the protein' calmodulin-dependent adenylate cyclase activity [3]. Within the intoxicated cell LF functions as a zinc-dependent metalloprotease and cleaves mitogen activated protein kinase kinases. LT can also activate the inflammasome in rodent models of intoxication [4]. Both toxins are lethal in animal models and cause a broad range of defects in target cells, including altered cell cycle, cell growth and survival, and attenuated inflammatory responses [5]. Collectively these activities of anthrax toxin cripple the host immune system and allow *B. anthracis* to grow to high numbers in the bloodstream [2], [6], [7], [8], [9]. Hence, immune neutralization of PA counters the damaging effects of anthrax toxin, providing protection to the host during early stages of disease.

PA-specific Ab neutralizes LT and ET in vitro and protects immunized animals in vivo following a lethal challenge with the toxins [10], [11], [12], [13], [14], [15]. There is a good correlation between PA-specific Ab titers and toxin neutralization by sera from patients who have survived *B. anthracis* infection [16]. Consequently, there is considerable interest in development of vaccines which incorporate PA as the immunogen but involves fewer immunizations, boosts immunological memory and prolongs neutralizing Ab production while stimulating a minimal inflammatory response [16], [17]. The current AVA anthrax vaccine administered to US military personnel consists of PA and induces PA-specific Ab titers sufficient to neutralize anthrax toxin [16], [17]. However, the Anti-PA Ab titers are not sustained and individuals require administration of multiple booster vaccines to maintain toxin-neutralizing Ab titers (http://www.anthrax.osd.mil/vaccine/schedule.asp#). Thus, there is a need for improving the efficacy of the current anthrax vaccine. Towards this effort, we recently demonstrated that activation of CD1d-restricted NKT cells with the CD1d-binding ligand (α-galactosylceramide, α-GC) at the time of immunization with PA led to enhanced and sustained Ab-mediated protection against anthrax lethal toxin [18]. Recent work by our laboratory and others has shown that NKT activation improves Ab responses against model and pathogen-derived Ags, suggesting that study of responses to anthrax could be useful for future application to other pathogens [19], [20], [21], [22], [23].

The mechanism by which α-GC-activated NKT cells provide help to PA-specific B cells is not clear, and whether α-GC represents the best choice of CD1d-binding adjuvant is also unknown. The α-GC ligand consists of a galactose headgroup with an α-anomeric linkage to hydrophobic sphingosine and acyl chains. The lipid moiety is loaded into hydrophobic pockets in the CD1d molecule expressed by professional Ag-presenting cells, orienting the galactose headgroup for recognition by NKT cells [24], [25]. The predominant NKT subset is represented by the Type I NKT cells which express an invariant Vα14, Jα18 TCR in mice and a Vα24, Jα28 TCR in humans [24], [25]. Type II NKT cells are CD1d-restricted, have variable Vα usage and are thought to be stimulated by a variety of self glycolipids rather than α-GC [24], [25].

Several researchers are investing considerable effort in designing α-GC-based molecules that have improved or more selective adjuvant effects than α-GC. Those focused on vaccines for cancer and malaria designed CD1d ligands that stimulate Th1-driven immune responses [26], [27]. The production of Th1 cytokines such as IFNγ by NKT cells and throughout the immune system is a reasonable indicator of how various CD1d ligands may function to impact Th1-driven immunity. In contrast, α-GC-related molecules have received little attention in the context of specific Ab responses that may be Th1 or Th2-driven.

We therefore examined the contribution of NKT cell-derived Th2 and Th1 cytokines to the production of PA-specific toxin-neutralizing Abs. Using IL-4^−/−^ and IFNγ^−/−^ mice, and bone marrow chimeric mice we demonstrate that a Th2-skewing CD1d ligand (OCH) modulates production of anthrax-toxin specific Ab subclasses. We show that the production of IgG1, IgG2b and IgG2c are affected by NKT-derived IL-4 and IFNγ. These findings provide much needed insight into a mechanism that can be exploited to optimize anthrax vaccination.

## Materials and Methods

### Reagents

BL21 competent cells were purchased from Invitrogen (Carlsbad, CA). The pET15b plasmids encoding PA and LF respectively were a gift from Dr. J. Collier (Harvard Medical School) and have been described previously [1]. The protein assay kit was purchased from Bio-Rad Laboratories (Hercules, CA). The Pyrogent Ultra Limulus Amebocyte Lysate kit was purchased from Lonza (Walkersville, MD). HRP-conjugated anti-IgG1 was purchased from Southern Biotechnology (Birmingham, AL). Fluorochrome-conjugated mAbs were purchased from BD Biosciences (San Jose, CA). The FcγR-blocking mAb (2.4G2) was purchased from BioXpres (West Lebanon, NH). CD1d tetramers (loaded with synthetic α-GC, PBS57) and the CD1d ligand OCH were provided by the NIAID Tetramer Facility (Emory University, Atlanta, GA). The α-GC ligand was purchased from Axorra Inc. (San Diego, CA).

### Toxin Expression and Purification

Histidine-tagged PA and LF were expressed separately in competent BL21 *Escherichia coli* (Invitrogen, Carlsbad, CA) transformed with the pET15b-rPA, pET15b-rLF and pET15brLF^H690C^ plasmids respectively. PA and LF were then purified from bacterial lysates using standard methods also described previously [28]. In brief, PA and LF purification was achieved using a 5 ml HisTrap FF affinity column (GE LifeSciences, Piscataway, NJ). LPS contamination was removed by from purified PA and LF using an EndoTrap Red LPS-binding affinity column (Lonza, Walkersville, MD). The concentration of residual endotoxin was determined using the Limulus Assay, which has a sensitivity of 0.06 Endotoxin Units/ml (6 pg/ml) (Lonza). The final preparation used for this study contained 40 ng/ml LPS in the PA and was below the limits of detection for the LF. No more than 4 ng LPS was administered to each mouse in experiments described herein. Of note, Histidine tags on anthrax toxin proteins did not induce anti-His Ab responses [29], [30].

### Mice

Female C57Bl/6 mice (CD45.2- and CD45.1-congenic) were purchased from the National Cancer Institute (Bethesda, MD). IL-4^−/−^ and IFNγ^−/−^ mice on a homogenous C57Bl/6 (CD45.2) genetic background were purchased from the Jackson Laboratory (Bar Harbor, ME). Jα18^−/−^ mice have a gene deletion in the TCR locus preventing rearrangement of the TCR gene specific to Type I NKT cells reactive to α-GC and related ligands [31]. Jα18^−/−^ mice were kindly provided by Dr. Kronenberg (La Jolla Institute for Allergy and Immunology, La Jolla, CA) following permission from Dr. Taniguchi (RIKEN Institute, Riken, Japan). The Jα18^−/−^ colony was maintained in a barrier facility at OUHSC. All experiments, unless indicated otherwise were commenced on mice at 6–10 wk of age.

### Ethics Statement

This study was carried out in strict accordance with the recommendations in the Guide for the Care and Use of Laboratory Animals of the National Institutes of Health. All animal procedures reported herein were approved by the University of Oklahoma Health Sciences Center Institutional Animal Care and Use Committee (IACUC). Protocol 10-085-HI was issued to authorize the experiments described herein. Unless indicated otherwise, procedures were performed under inhalational isofluorane anesthesia (96% oxygen, 4% isofluorane), and all efforts were made to minimize suffering. Procedure associated-deaths precluded the use of anesthesia for in vivo lethal challenge assays. This method was approved by the IACUC and performed by a qualified veterinarian (S.K. Joshi DVM).

### Isolation of splenocytes and bone marrow

Spleens were harvested into RPMI buffer and a single cell suspension obtained by mechanical disruption. Bone marrow cells were flushed from the femurs and tibias using a 27 gauge needle and syringe filled with media. Erythocytes were removed by incubation with ammonium chloride lysis buffer (0.16 M NH_4_Cl, 0.17 M Tris-HCl, pH 7.4) for 2 min at 37°C. After washing in culture media, cell viability was confirmed as >98% by trypan blue exclusion. Cells were enumerated using a Nexelom cell counter (Lawrence, MA).

### Bone Marrow Chimeras

Naïve 6 wk old female C57Bl/6 CD45.1 congenic mice were irradiated (700 Rad) and rested for 18 h before a second irradiation (500 Rad). After a further 4 h, donor bone marrow cells were transferred by the i.v. route to the irradiated recipients. One million donor cells were transferred and all donor mice expressed the CD45.2 allele. Donor bone marrow consisted of the following 50∶50 mixtures: Jα18^−/−^/C57Bl/6; Jα18^−/−^/IL-4^−/−^; Jα18^−/−^/IFNγ^−/−^. Recipient mice were then housed for a period of 12 wk. Mice were either euthanized or immunized and subsequently bled as described. Euthanized mice were used as a source of cells for analysis of re-engraftment of the bone marrow, re-constitution of the lymphoid and myeloid compartments in the periphery, and in vitro functional assays.

### Immunizations and experimental schedule

Female mice of 6–10 wk of age were used and five mice per group were immunized unless indicated otherwise. A single subcutaneous (s.c.) immunization was administered over both flanks on d 0 immediately following collection of pre-bleed sera. Unless indicated otherwise, immunizations consisted of 10 µg PA in 200 µl sterile-endotoxin-free PBS or PA mixed with 4 µg of OCH. Mice were then bled at d 14 post-immunization and sera obtained. On d 28 mice were bled and then boosted s.c. with 5 µg of PA in PBS and bled on d 45 and between d 110–120 unless indicated otherwise. At the end of the experimental period, mice were either challenged with toxin or euthanized in order to obtain bone marrow and spleen.

### Retro-orbital eye bleed and serum collection

Mice were anesthetized using a vaporized 4% isofluorane/96% oxygen mixture and 100 µl blood collected by retro-orbital bleed with heparinized micro-capillary tubes (Fisher Scientific, Hampton, NH). Samples were transferred immediately to polypropylene micro-centrifuge tubes. Blood samples were incubated for 30 min at room temperature then allowed to clot overnight at 4°C, before centrifugation at 13,000 *g* for 15 min at 4°C. Sera were withdrawn with a pipette and stored in aliquots at −20°C.

### Passive transfer Experiments

C57Bl/6 mice were immunized s.c. with 10 µg PA on d 0 and boosted with PA on d 28. Sera were collected every two d starting on d 38 and ending on d 48. Sera from different time points and the different mice within the same group were then pooled. Sera were also collected from naïve mice and pooled. A five hundred microliter volume of sera was then transferred by the i.p. route to recipient IL-4^−/−^ mice. After further 24 h of incubation, mice were challenged with LT.

### *In Vivo* Toxin Challenge

The LF and PA subunits were mixed at a 1∶1 molar ratio in PBS. An amount of lethal toxin equivalent to 200 µg PA and in a 100 µl volume was then administered by the i.v. paraorbital route to mice. After 24 h a second dose equivalent to 100 µg PA was administered. The mice were then monitored daily for the duration of the experiment and time to death or euthanasia was recorded by a qualified DVM. Pain and suffering were minimized as follows: The mice were housed with food and water ad libitum with a 12 hr light/dark cycle; Mice were kept in groups of 5 in their own cage throughout the experiments; If mice were observed to have progressed to hunched posture, ruffled fur, self-imposed isolation, slow movement and tremors, they were euthanized. Toxin administration is typically associated with few immediately visible effects in C57Bl/6 mice and is followed by rapid worsening and death. Otherwise, death was the experimental endpoint. A Log-rank Mantel-Cox test in conjunction with Kaplan-Meier survival curves was used to measure differences between experimental groups.

### ELISA for Serum Ig

Immulon 4 ELISA–plates (Dynex Technologies Inc. Chantilly, VA), were coated with PA at 10 µg/ml in binding buffer (0.1 M Na_2_HPO_4_, pH 9.0), overnight at 4°C before washing plates and blocking for 2 h at room temperature with 1.0% w/v BSA in PBS/0.05% v/v Tween 20. Sera were diluted 100 or 10,000 fold in PBS/0.05% v/v Tween and subjected to two-fold serial dilutions, before adding to PA-coated, pre-blocked plates. Plates were incubated overnight at 4°C with diluted sera, before washing 4 times in PBS/0.05% v/v Tween 20. Plates were incubated for 1 h at room temperature with HRP-conjugated anti-mouse IgG1, IgG2b or IgG2c at a final concentration of 0.2 µg/ml. Plates were washed and developed for 8 min at room temperature using 90 µl of ABTS substrate per well (KPL, Gaithersburg, MD). Reactions were stopped by addition of 110 µl of a 10% w/v SDS solution. Plates were analyzed using a Dynex MRX Revelation plate reader. Endpoint titers were determined as O.D. <0.01 at 405 nm (equivalent to O.D. of 1/200 dilution of pre-bleed sera or two-times background). Individual Ab titers were plotted as geometric means using GraphPad Prism software. A non-parametric Mann-Whitney U test was used to assess experiments with two experimental groups. Where titers were not detectable, a value of 100 was assigned corresponding to the highest concentration of serum in the assay. Multiple experimental groups were assessed by one-way ANOVA with Dunn's post-test.

### *In Vitro* Toxin Neutralization Assay

Murine 264.7 macrophages were adjusted to 10^6^ cells/ml in DMEM containing 4 µM L-glutamine, 10% FCS and 40 µg/ml gentamicin. One hundred thousand cells in a 100 µl volume were then seeded into 96 well culture plates and incubated overnight with 5% CO_2_. Serial two-fold dilutions of sera in media were made (1/125–1/500), mixed with lethal toxin at a final concentration of 1–16 µg/ml of each subunit. The toxin mixture was then incubated for 1 h at room temperature. Media were then removed from cell cultures and replaced with the sera/toxin/media mixture. Plates were incubated for a further 2 h at 37°C before addition of Cell counting Kit-8 (Dojindo Technologies, Rockville, MD). After 2 h incubation, plates were read at an absorbance of 450 nm. Differences in Ab titers between two experimental groups were assessed for statistical significance using a non-parametric Mann-Whitney U test. As with ELISA experiments the Mann Whitney U test was used because an independent control was performed for each experimental group.

### Flow Cytometry

Cells were incubated at 4°C or room temperature at a density of 10^7^ cells/ml in RPMI plus 10% FCS with 2.4G2 mAb at a final concentration of 20 µg/ml. Fluorochrome-conjugated mAbs were added at a 1∶100 to a 1∶500 dilution as appropriate or with APC-conjugated CD1d tetramer at a 1∶250 dilution. After 1 h unbound mAb was removed by washing and centrifugation. Cells were fixed with 1% w/v para-formaldehyde and analyzed using a Becton-Dickinson FACSCalibur (Palo Alto, CA).

### Cytokine Assays

Mice were immunized i.p. with 4 µg α-GC or 4 µg OCH in a 100 µl volume of PBS/0.05% v/v Tween 20. Sera were collected before immunization and at 6 and 22 h thereafter. Samples were analyzed using an IFNγ or IL-4 sandwich ELISA in accordance with the manufacturer's instructions (BD Biosciences, Mountain View, CA) and as described previously [32].

## Results

### Requirement for IL-4 but not IFNγ in the production of lethal toxin neutralizing Ab

**Figure 1:**
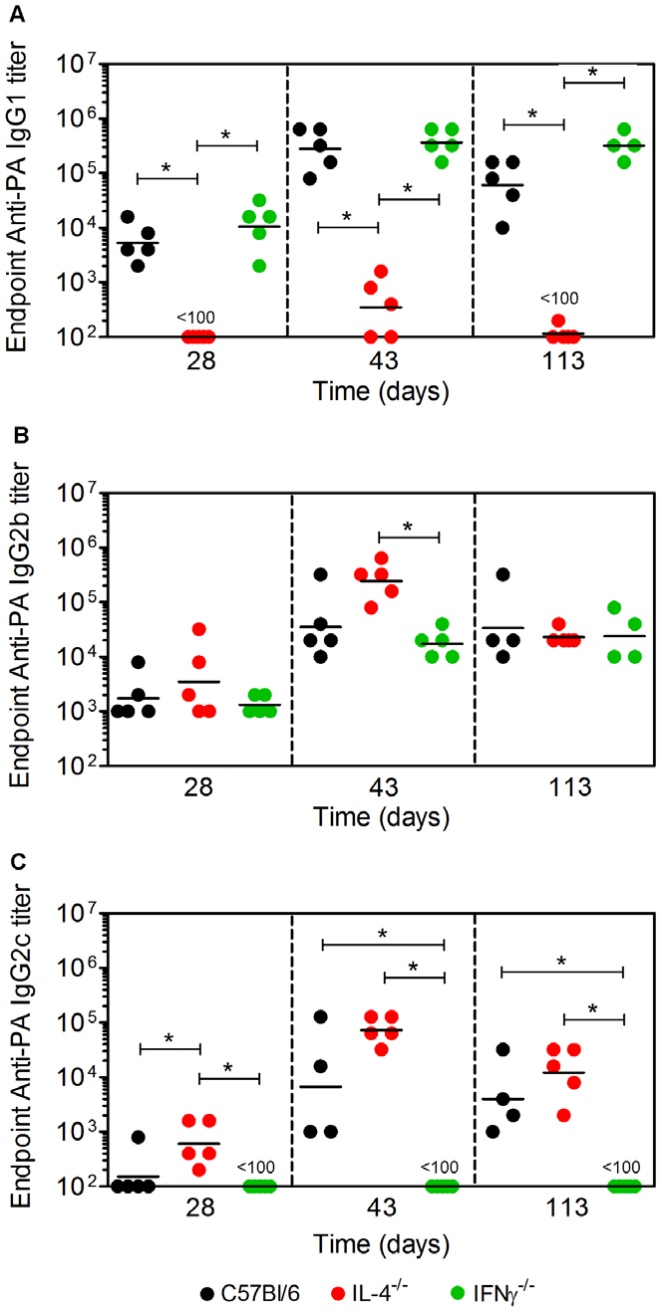
Requirement for IL-4 and IFNγ in production of PA-specific IgG1 or IgG2c. C57Bl/6, IL-4^−/−^ and IFNγ^−/−^ mice were immunized and bled as described. **A** Endpoint anti-PA IgG1, **B** IgG2b, and **C** IgG2c titers were determined by ELISA. Each data point represents an individual mouse and geometric mean titers are shown. Data are representative of three independent experiments. Statistical significance (p<0.05) is indicated by asterisks.

Experiments were performed to determine which Ab subclasses accounted for protection against LT in vivo in the C57Bl/6 mouse strain. C57Bl/6 control mice and IL-4^−/−^ and IFNγ^−/−^ mice on the C57Bl/6 genetic background were immunized s.c. with 10 µg PA on d 0 and boosted with 5 µg of PA on d 28. Blood samples were collected and sera prepared on d 28, 42 and 113, previously established as appropriate times to measure PA-specific short-term primary, recall, and long-term Ab titers [33] (Figure 1). Endpoint anti-PA IgG1, IgG2b and IgG2c titers were determined by ELISA (Figure 1A–C). IgG2c rather than IgG2a was assayed because the γ2a gene segment is deleted in the C57Bl/6 genetic background [34]. In C57Bl/6 and IFNγ^−/−^ mice, IgG1 was the predominant Ab subclass produced. Titers were higher following the booster vaccine than in primary sera and were sustained over the course of the experiment. In IL-4^−/−^ mice, there was a marked absence of IgG1 production, but high IgG2b and IgG2c titers were observed. These data show as expected that IL-4 was required for production of anti-PA IgG1. In contrast, IFNγ was dispensable for IgG1 production, but drove IgG2b and IgG2c production in the absence of IL-4.

**Figure 2:**
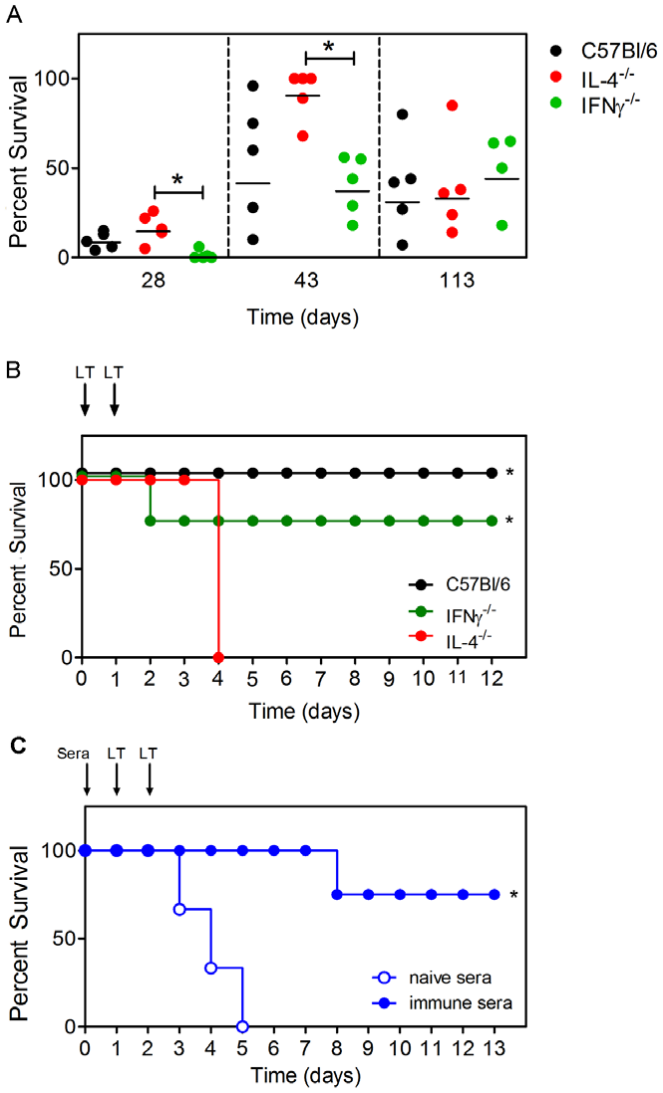
Requirement for IL-4 but not IFNγ in production of PA-neutralizing IgG. **A** Sera from experiment in Figure 1 were tested for their ability to protect RAW267.4 macrophages from LT toxicity. Graph shows neutralization by sera from C57Bl/6 mice, IL-4^−/−^ and IFNγ^−/−^ mice. Each data point represents an individual mouse and the mean survival of the cell line following treatment with LT and sera is indicated. **B** Mice were challenged with LT as indicated. **C** Pooled sera from naïve (n = 5) and PA-immunized (n = 5) mice were transferred to naïve IL-4^−/−^ mice before challenge with LT. Three and four IL-4^−/−^ mice per group received naïve and immune sera respectively. Data in A and B are representative of two independent experiments while data in C are from a single experiment. Graphs show percent survival following LT administration. Statistical significance (p<0.05) is indicated by asterisks.

Sera from immunized C57Bl/6, IL-4^−/−^ and IFNγ^−/−^ mice were then used to determine the ability of anti-PA Ab to neutralize LT in vitro (Figure 2A). In each of the three mouse strains used the neutralization capacity corresponded to the Ab titers regardless of subclass, showing that a combination of: IgG1, IgG2b and IgG2c; IgG2b and IgG2c; or IgG1 and IgG2b were capable of neutralizing LT in vitro. The immunized C57Bl/6, IL-4^−/−^ and IFNγ^−/−^ mice were also administered a lethal dose of LT by the i.v. route on d 115 and survival was monitored over a 12 d period (Figure 2B). C57Bl/6 and IFNγ^−/−^ mice showed a transient worsening in health over 48 h followed by recovery, and ultimately survived the challenge. In contrast, all IL-4^−/−^ mice displayed rapidly worsening health, and died within 96 h of the challenge. These data show that while sera containing IgG1, IgG2b and IgG2c, IgG1 and IgG2b or containing IgG2b and IgG2c adequately neutralized LT in vitro, the latter did not confer adequate protection in vivo.

Since death in the IL-4^−/−^ mice could have been attributed to a defect other than lack of IgG1, sera were collected from naïve and PA-immunized C57Bl/6 (containing IgG1, IgG2b and IgG2c) and transferred to naïve IL-4^−/−^ mice before LT challenge (Figure 2C). In that experiment, 75% of mice that received sera from the PA-immunized mice survived. In contrast 0% of the mice that received sera from the naïve mice survived, confirming that lack of survival of IL-4^−/−^ mice was attributable to a lack of PA-specific IgG1 rather than another IL-4-associated defect.

### Effect of Th2-polarizing CD1d ligand OCH on production of lethal toxin-neutralizing IgG1

**Figure 3:**
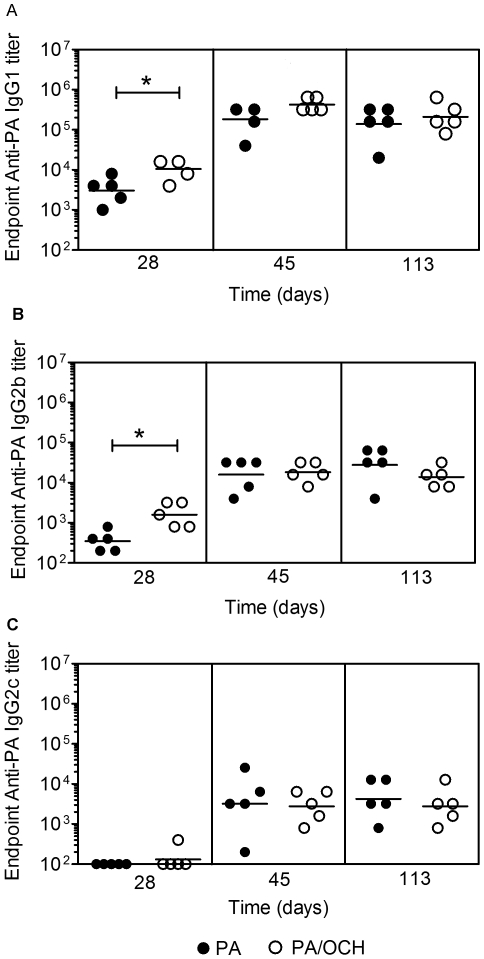
Effect of OCH on PA-specific IgG1, IgG2b and IgG2c response. C57Bl/6 mice were immunized and bled as described. **A** Endpoint anti-PA IgG1, **B** IgG2b and **C** IgG2c titers were determined by ELISA. Each data point represents an individual mouse (5 per group) and geometric mean titers are shown. Data are representative of two independent experiments. Statistical significance (p<0.05) is indicated by asterisks.

It was reported previously that the CD1d ligand α-GC which stimulates a mixed Th1/Th2 response in vivo, led to production of PA-specific, IgG1, IgG2b and IgG2c, albeit dominated by the IgG1 subclass [33]. In this study, IgG1, IgG2b and IgG2c-containing sera were more protective in vivo than sera containing IgG2b and IgG2c, implicating IgG1 as a good correlate of in vivo protection. C57Bl/6 mice were therefore immunized with PA or PA plus OCH (an α-GC variant). OCH has weaker activity than α-GC and skews NKT cells to Th2 cytokine production [35], [36]. PA/OCH administration was followed by a booster vaccine consisting of PA. Sera were collected before (d 28) and after (d 45 and 113) the booster and PA-specific endpoint IgG1, IgG2b and IgG2c titers determined by ELISA. OCH enhanced the anti-PA IgG1 and IgG2b responses measured on d 28, but had no measurable effect on longer-term titers measured at d 45 and 113 (Figure 3A–B). OCH had no effect on IgG2c responses measured at d 28, d 45 or d 113 (Figure 3C). As a further control C57Bl/6 mice were immunized with vehicle, α-GC or OCH, before collecting sera and measuring IL-4 and IFNγ production. The selective production of IL-4 in the C57Bl/6 environment following OCH administration confirmed that the response to OCH was Th2-polarized (Figure S1). Therefore the result show that a Th2-skewing OCH boosted toxin-specific primary IgG1 and IgG2b responses in C57Bl/6 mice but did not offer the sustained response previously observed for the α-GC ligand [18]. This suggests that a Th2-skewing CD1d ligand can accelerate the primary IgG1 and IgG2b response but does not increase the ultimate Ab titer following a booster vaccine.

### Effect of OCH on IgG1, IgG2b and IgG2c production in IL-4^−/−^ mice

**Figure 4:**
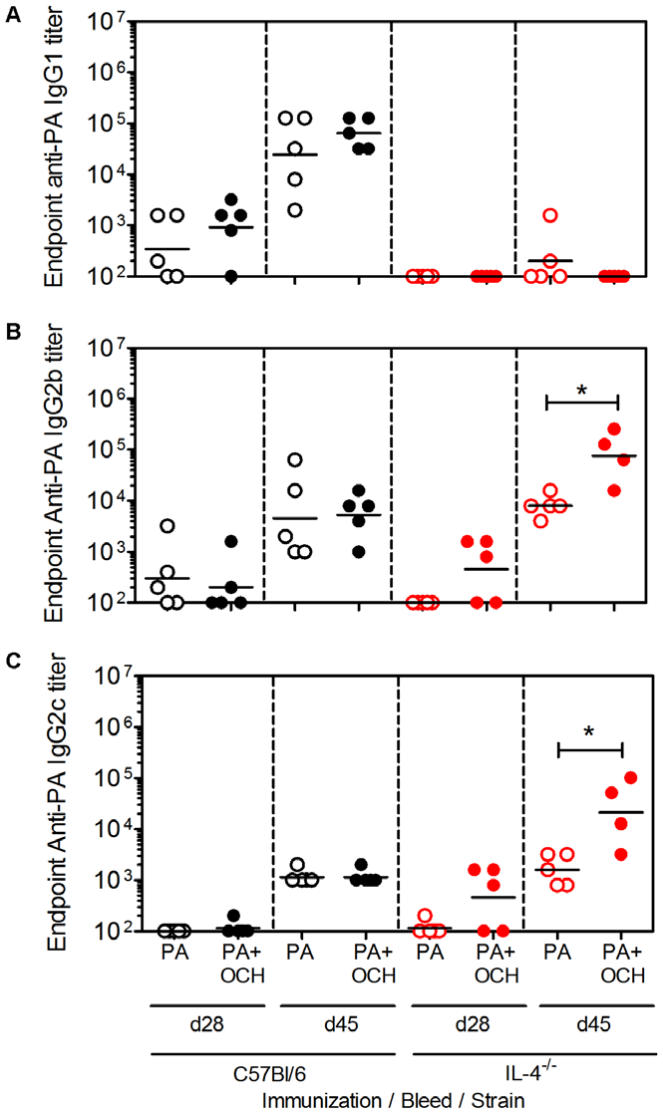
OCH-enhanced IgG2b and IgG2c responses in IL-4^−/−^ mice. C57Bl/6 and IL-4^−/−^ mice were immunized and bled as described in materials and methods. **A** Shows endpoint anti-PA IgG1 titers. **B** Shows endpoint anti-PA IgG2b titers. **C** Shows endpoint anti-IgG2c titers. Each data point represents an individual mouse (4 or 5 per group as indicated) and geometric mean titers are shown. Data are representative of two independent experiments.

C57Bl/6 and IL-4^−/−^ mice were immunized and bled as described in materials and methods, except that the experiment was shortened to duration of 45 days. This permitted toxin challenge when IgG2b and IgG2c titers were highest. In C57Bl/6 mice, OCH had a similar effect on the d 45 IgG1, IgG2b and IgG2c titers as compared to the previous experiment in Figure 3, although the adjuvant effect was less apparent in d 28 bleeds (Figure 4A–C). OCH did not induce measurable IgG1 responses in IL-4^−/−^ mice, but did significantly boost IgG2b and IgG2c titers. This showed that OCH-stimulated IL-4^−/−^ NKT cells were capable of boosting IgG2b and IgG2c responses. Further examination of the sera using ELISA plates coated with high versus low concentrations of PA, revealed that OCH did not affect the overall affinity of PA-specific IgG1, IgG2b or IgG2c in C57Bl/6 or IL-4^−/−^ mice (Figure S2).

**Figure 5:**
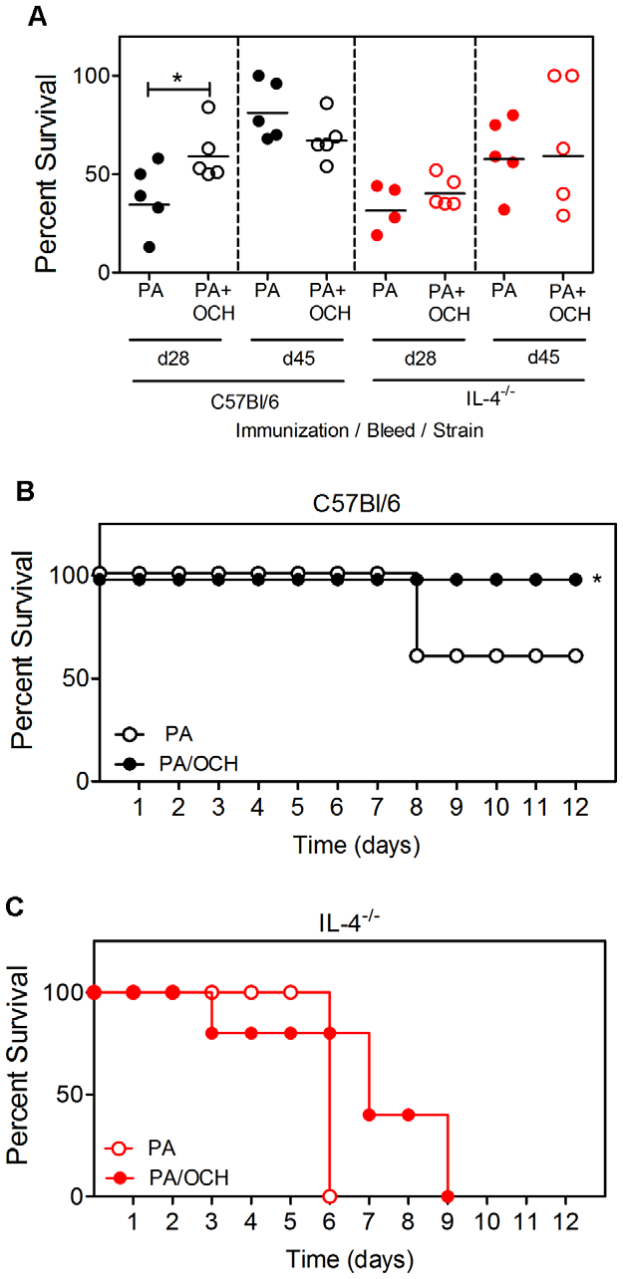
Non-protective OCH-enhanced IgG2b and IgG2c responses in IL-4^−/−^ mice. **A** Sera from mice in Figure 4 were tested for neutralization of LT in vitro. **B** C57Bl/6 and **C** IL-4^−/−^ mice were challenged as indicated. Toxin was administered on d 50 when IgG2 titers were highest. Graphs show percent survival following LT administration. Statistical significance (p<0.05) is indicated by asterisks. Each data point in A represents an individual mouse (4 or 5 per group as indicated). All mice in A were challenged for parts B and C. Data are representative of two independent experiments.

Sera from immunized C57Bl/6 and IL-4^−/−^ mice were then tested for neutralization of anthrax toxin in vitro (Figure 5A). OCH led to an enhanced in vitro neutralization by d 28 sera from C57Bl/6 mice but not IL-4^−/−^ mice. On subsequent bleeds (d 45) OCH did not have a measurable effect on neutralization. As in Figure 2, sera from immunized C57Bl/6 and IL-4^−/−^ mice had comparable in vitro neutralization. The immunized C57Bl/6 and IL-4^−/−^ mice were challenged with LT (on d 48). Survival was monitored daily (Figure 5B, C). C57Bl/6 mice immunized with PA alone had a 60% survival rate following toxin challenge, whereas 100% of mice immunized with PA plus OCH survived. Although modest, the differences were statistically significant (Figure 5C). This is consistent with the Ab titer and neutralization data shown in Figure 3 and 4, showing that OCH has a moderate effect on protective IgG1 responses. In contrast, PA-immunized IL-4^−/−^ mice all died within 6 d of toxin challenge, consistent with results in Figure 1. IL-4^−/−^ mice immunized with PA plus OCH died steadily in the days following challenge and had a 0% survival rate over 9 d that did not differ significantly from the control group (Figure 5C). These results showed that a Th2-skewing CD1d ligand could stimulate a Th1 Ab response in the absence of IL-4.

### Modulation of PA-specific IgG1, IgG2b and IgG2c by NKT-derived IL-4 and IFNγ

**Figure 6:**
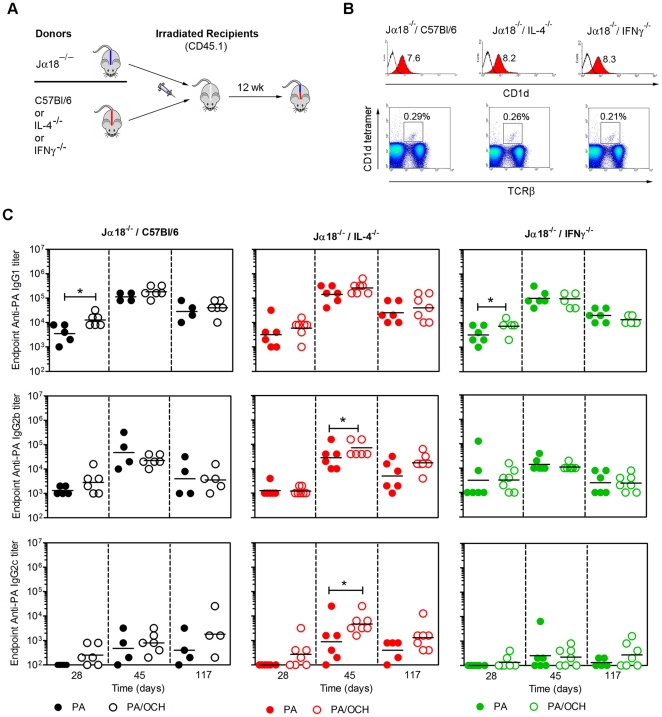
Modulation of PA-specific IgG1, IgG2b and IgG2c by NKT-derived IL-4 and IFNγ. **A** Bone marrow chimeras were generated as indicated and detailed in materials and methods. **B** Splenocytes from chimeras were analyzed by flow cytometry for expression of CD1d (histograms) and re-constitution of CD1d-tetramer^+^/TCRβ^+^ NKT cells (dot plots). Mean fluorescent intensity for anti-CD1d mAb binding and percent frequency of CD1d-tetramer^+^/TCRβ^+^ cells are indicated. Comparable numbers of total splenocytes were recovered from each chimera indicating comparable absolute numbers of NKT cells. Reconstitution of cell populations and NKT cells has been demonstrated in at least 6 independent experiments. **C** Chimeric mice were immunized and bled as described. Top row shows endpoint anti-PA IgG1 titers on the days indicated following immunization. Middle row shows endpoint anti-PA IgG2b titers. Bottom row shows endpoint anti-IgG2c titers. Each data point represents an individual mouse (5–7 per group as indicated). Data are representative of two similar independent experiments (except for α-GC as adjuvant). Statistical significance (p<0.05) is indicated by asterisks. Further statistical comparisons (ANOVA) were employed to compare the d45 bleeds for IgG2b and IgG2c between the three different chimeras. The IgG2b and IgG2c titers were significantly lower in the Jα18^−/−^/IFNγ^−/−^ chimeras than the Jα18^−/−^/IL-4^−/−^ chimeras but not the Jα18^−/−^/C57Bl/6 chimeras (not depicted on figure).

Bone marrow chimeras were constructed in which NKT cells could or could not express IL-4 or IFNγ while other immune cells retained the capacity for IL-4 and IFNγ production (Figure 6). Three different chimeras were constructed whereby irradiated recipient mice were engrafted with a 50∶50 mix of donor bone marrow from Jα18^−/−^ mice and C57Bl/6 mice (Jα18^−/−^/C57Bl/6), with a 50∶50 mix from Jα18^−/−^ mice and IL-4^−/−^ mice (Jα18^−/−^/IL-4^−/−^) or with a 50∶50 mix from Jα18^−/−^ mice and IFNγ^−/−^ mice (Jα18^−/−^/IFNγ^−/−^) (Figure 6A). All three chimeras were equivalently reconstituted with major immune cell types that were donor-derived (Figure S3) and had equivalent expression of CD1d and re-constitution of the NKT compartment (Figure 6B).

Jα18^−/−^/C57Bl/6, Jα18^−/−^/IL-4^−/−^ and Jα18^−/−^/IFNγ^−/−^ chimeras were immunized with PA alone or PA plus OCH and later boosted with PA alone (Figure 6C). Primary and recall sera were collected and a contribution of NKT-derived IL-4 was apparent for the d 28 IgG1 response, but not for the d 45 and 117 responses. Absence of NKT-derived IFNγ had no discernable effect on IgG1 titers. These data indicate that NKT-derived IL-4 accelerated the production of PA-specific IgG1. In the Jα18^−/−^/IL-4^−/−^ chimeras, IgG2b and IgG2c were boosted by OCH. In contrast, OCH had no measurable adjuvant effect on IgG2b and IgG2c in the Jα18^−/−^/IFNγ^−/−^ chimeras (Figure 6C). These results show that NKT cell-derived IL-4 accelerates PA-specific IgG1 responses, but suppresses PA-specific IgG2c responses. In contrast, NKT-derived IFNγ is dispensable for IgG1 production but contributory to the IgG2b and IgG2c responses.

We performed experiments to measure in vitro LT neutralization by sera obtained from the chimeric mice (Figure S4). We observed that all sera from the d 45 bleeds exhibited neutralization of LT in vitro. There was apparent variance in neutralization between experimental groups but comparison between PA- and PA/OCH-immunized groups or between the chimeras did not show statistically significant differences. The PA/OCH-immunized mice were challenged with LT 120 d after immunization. All groups succumbed with a similar time-course and all mice died within 6 d of LT administration, suggesting that bone marrow chimeric mice are too susceptible to LT for the challenge experiments routinely carried out on the C57Bl/6 background (*data not shown*).

## Discussion

The objective of this study was to identify critical elements of NKT-dependent vaccination against anthrax toxin and use this information to improve production of neutralizing antibodies targeting PA. To achieve this goal, it was necessary to revisit the question of which murine IgG subclasses confer protection against anthrax toxin. A number of studies have shown that following PA immunization, IgG1, IgG2 and IgG3 subclasses can all be observed [37]. Studies using mAbs of known subclass have elegantly demonstrated that IgG1 or IgG2a can be harmful or beneficial to macrophages in vitro by affecting cellular entry of toxin [38]. It is therefore possible, perhaps likely, that harmful and beneficial Abs of all subclasses are formed during an in vivo polyclonal response to immunization. We therefore obtained IL-4^−/−^ mice and IFNγ^−/−^ mice on the C57Bl/6 genetic background. These strains are well known for their poor capacity to stimulate production of IgG1 and IgG2c respectively [39], [40], and it was therefore expected that they would be unable to produce PA-specific IgG1 or IgG2c (as was observed). The sera from immunized C57Bl/6, IL-4^−/−^ and IFNγ^−/−^ mice were all able to neutralize PA in vitro but sera containing IgG2b and IgG2c was at least as protective in vitro as sera containing IgG1, IgG2b and IgG2c or containing IgG1 and IgG2b.

For reasons that are presently unclear OCH boosted primary IgG1 responses in C57Bl/6 mice, but only secondary responses in IL-4^−/−^ mice. This suggests NKT-derived IL-4 may boost the primary response and suppress the memory response induced by OCH. Exploration of this concept would require adoptive transfer approaches using congenic recipients to delineate between primary and recall Ab titers in the strains used and is warranted in future studies.

The in vitro neutralization assays contrasted with the in vivo challenge experiments because IL-4^−/−^ mice lacking anti-PA IgG1 were not protected against LT. In that experiment, IFNγ^−/−^ mice with IgG1 and IgG2b but no IgG2c were protected similarly to C57Bl/6 controls containing IgG1, IgG2b and IgG2c. These results therefore suggest the possibility that a polyclonal PA-specific IgG1 response is more protective in vivo than a polyclonal IgG2b or IgG2c response. This was confirmed by transfer of sera PA-immunized C57Bl/6 mice into naïve IL-4^−/−^ recipients, whereby protection was conferred. The reason for the lack of protection conferred by IgG2b and IgG2c in the IL-4^−/−^ mice is presently unclear. Arguably, the absolute amount of IgG2b and IgG2c titers in immunized mice could have been insufficient to neutralize toxin. However, IgG2b titers at the time of in vivo toxin challenge in PA/OCH-immunized IL-4^−/−^ mice were comparable to IgG1 titers in PA/OCH-immunized C57Bl/6 mice and three-fold higher than in PA-immunized C57Bl/6 mice (Figure 4). Under these conditions, the IL-4^−/−^ mice were still less protected than the PA- or the PA/OCH-immunized C57Bl/6 mice. Given that the different IgG sub-classes were detected using the same methods and reagents (with the exception of the detection Ab), then it is not highly likely that the amount of IgG2b and IgG2c was too low to be protective.

An alternative explanation is that collectively IgG1, IgG2b and IgG2c are required for protection, perhaps by invoking a range of effector mechanisms, however, IFNγ^−/−^ mice lacked IgG2c responses and displayed comparable neutralization and survival as the C57Bl/6 controls. Another potential explanation is that the different cytokine environments affected the specificity of the anti-PA IgG responses. However, in IFNγ^−/−^ mice we did not observe changes in protection. Arguably Ab specificities could be different in IL-4^−/−^ mice, but with a polyclonal response, this does not appear likely. Epitope mapping studies would be required to address this hypothesis.

It was recently found that mAbs with the same PA-specificity recombined with IgG1, IgG2a and IgG2b constant regions were all protective in vivo (when the same dose was administered) and that IgG2a was superior to IgG1. Furthermore, the protection was FcγR dependent [41]. It is difficult to directly compare the results of that study with our findings, since that study compared IgG2a to IgG1 and passive transfer of monoclonal responses was examined. Arguably by transferring IgG2a into a mouse strain that does not normally express it, the IgG2a subclass may have had an exaggerated protective effect in vivo due to lack of competition for FcR binding by other IgG2a molecules. Equally possible however, is that IgG1 could have assumed a more exaggerated role in our experiments (as compared to IgG2b and IgG2c) because IgG2a was absent. Clearly, neither of these studies represents the last word in regard to which Ab classes are optimal for protection, but indicate that there is considerable complexity in the neutralizing Ab response that warrants further analysis.

Given that sera containing IgG1, IgG2b and IgG2c or containing IgG1 and IgG2b was more protective than serum containing IgG2b and IgG2c in vivo, we tested a Th2 cytokine-polarizing CD1d ligand known as OCH [36]. OCH is similar in structure to the better known α-GC molecule that stimulates a mixed Th1/Th2 response, except that it has a truncated sphingosine chain and a slightly shortened acyl chain. We observed that OCH enhanced the primary IgG1 response, and to a lesser extent the recall IgG1 response, but did not polarize Ab production away from IgG2b or IgG2c. Consequently, there was little difference between PA and PA/OCH-immunized C57Bl/6 mice as regards in vitro neutralization or in vivo protection. Furthermore, OCH was unable to overcome the lack of IgG1 production in IL-4^−/−^ mice, but significantly boosted IgG2b and IgG2c titers. These experiments suggested a functional dissociation between the reported mechanisms of action of OCH (IL-4 production by NKT cells) and its effects on an NKT-enhanced Ab response. We therefore conducted experiments to ask directly whether NKT-derived IL-4 or IFNγ had any influence on the anti-PA Ab profile. Bone marrow chimeras whereby all NKT cells were able to express IFNγ and IL-4, IFNγ only or IL-4 only were created, immunized and bled. The data revealed that NKT-derived IL-4 accelerated the production of anti-PA IgG1. This was evident in the reduced primary IgG1 responses following PA/OCH administration in chimeras lacking NKT-derived IL-4.

The absence of NKT-derived IL-4 did not substantially impact recall IgG1 titers. It is noteworthy that in the chimeras, we have frequently observed that OCH (and α-GC) do not lead to as great an enhancement of recall Ab responses as in C57Bl/6 mice. This experimental system could therefore not be used to rule out effects of IL-4 on recall IgG1 responses. An alternative explanation is that other NKT-derived Th2 cytokines such as IL-13 compensate for the lack of IL-4. However, in IL-4^−/−^ mice, NKT cell stimulation was unable to generate an IgG1 response.

NKT-derived IL-4 and IFNγ also affected IgG2b and IgG2c production. In the presence of IL-4^−/−^ NKT cells, there was an enhancement of the anti-PA IgG2b and IgG2c responses. In contrast, there was a near absence of OCH adjuvant effect on anti-PA IgG2b and IgG2c in the presence of IFNγ^−/−^ NKT cells. These results suggest that one function of NKT-derived IFNγ might be to boost production of less abundant Ab subclasses, whereas IL-4 may suppress that response.

We have therefore shown in this study that NKT-derived cytokines can exert modulating effects on toxin-neutralizing Ab responses, but have differential impacts on distinct Ab subclasses. Our results may have impact in two areas. Firstly, assessing IL-4 and IFNγ production by NKT cells in response to stimulation with various CD1d ligands may not be a sufficient indicator of the ensuing immune response, particularly with regards to Th2-driven humoral responses. Secondly, vaccination strategies that seek to harness NKT activation may need to take account of the Ab subclasses that are beneficial or harmful against a given pathogen.

## Supporting Information

Figure S1 **Polarized Th2 response in mice stimulated with OCH.** C57Bl/6 mice were immunized i.p. with vehicle (PBS/Tween), vehicle plus α-GC, or vehicle plus OCH. Sera were collected prior to immunization and at 6 and 22 h thereafter. Concentration of IL-4 and IFNγ in the samples was then determined by sandwich ELISA. Data shows mean cytokine concentration for 3 (α-GC) and 5 (OCH) mice per group. CD1d^−/−^ were also treated but did not elicit any measurable response to α-GC or OCH (not depicted).
(TIF)

Figure S2 **Affinity of PA-specific IgG1, IgG2b or IgG2c are not affected by OCH or by lack of IL-4.** In vivo d 45 sera from experiment reported in Figure 4 were assessed by ELISA. Plates were coated with PA at a final concentration of 2 μg/ml and 20 μg/ml respectively. Sera at a 1/1000 dilution were then incubated on the plate with both PA concentrations before detection of Ab sub-classes. Bar graphs indicate the mean ±SD ratio of the A405 for samples applied to the 2 μg/ml wells versus the 20 μg/ml wells indicating the proportion of high affinity Ab in the samples.
(TIF)

Figure S3 **Effective reconstitution of the hematopoetic compartment following lethal irradiation.** Spleens were obtained from immunized Jα18^−/−^/C57Bl/6, Jα18^−/−^/IL-4^−/−^ and Jα18^−/−^/IFNγ^−/−^ chimeric mice and analyzed by flow cytometry. Top row shows CD45.2^+/+^ donor cells and residual CD45.1^+/+^ recipient cells in re-constituted mice. Second row shows re-constitution with donor-derived T cells. Third row shows re-constitution with donor-derived B cells. Fourth row shows re-constitution with donor-derived dendritic cells.
(TIF)

Figure S4 **In vitro neutralization of lethal toxin by sera from PA-immunized chimeric mice.** The d 45 sera from the experiment in Figure 6 were tested for their ability to protect RAW267.4 macrophages from LT toxicity. Graph shows neutralization by sera from Jα18^−/−^/C57Bl/6, Jα18^−/−^/IL-4^−/−^ and Jα18^−/−^/IFNγ^−/−^ chimeras. Each data point represents an individual mouse and the mean survival of the RAW267.4 macrophages following treatment with LT and sera is indicated. One of the sera from the Jα18^−/−^/C57Bl/6 group immunized with PA was not analyzed due to an insufficient amount of sample remaining.
(TIF)
