# Supplementary figures and images for: Regulation of Anthrax Toxin-Specific Antibody Titers by Natural Killer T Cell-Derived IL-4 and IFNγ
Source: PLoS One. 2011 Aug 17;6(8):e23817. doi: 10.1371/journal.pone.0023817 (PMC3157475; doi:10.1371/journal.pone.0023817)

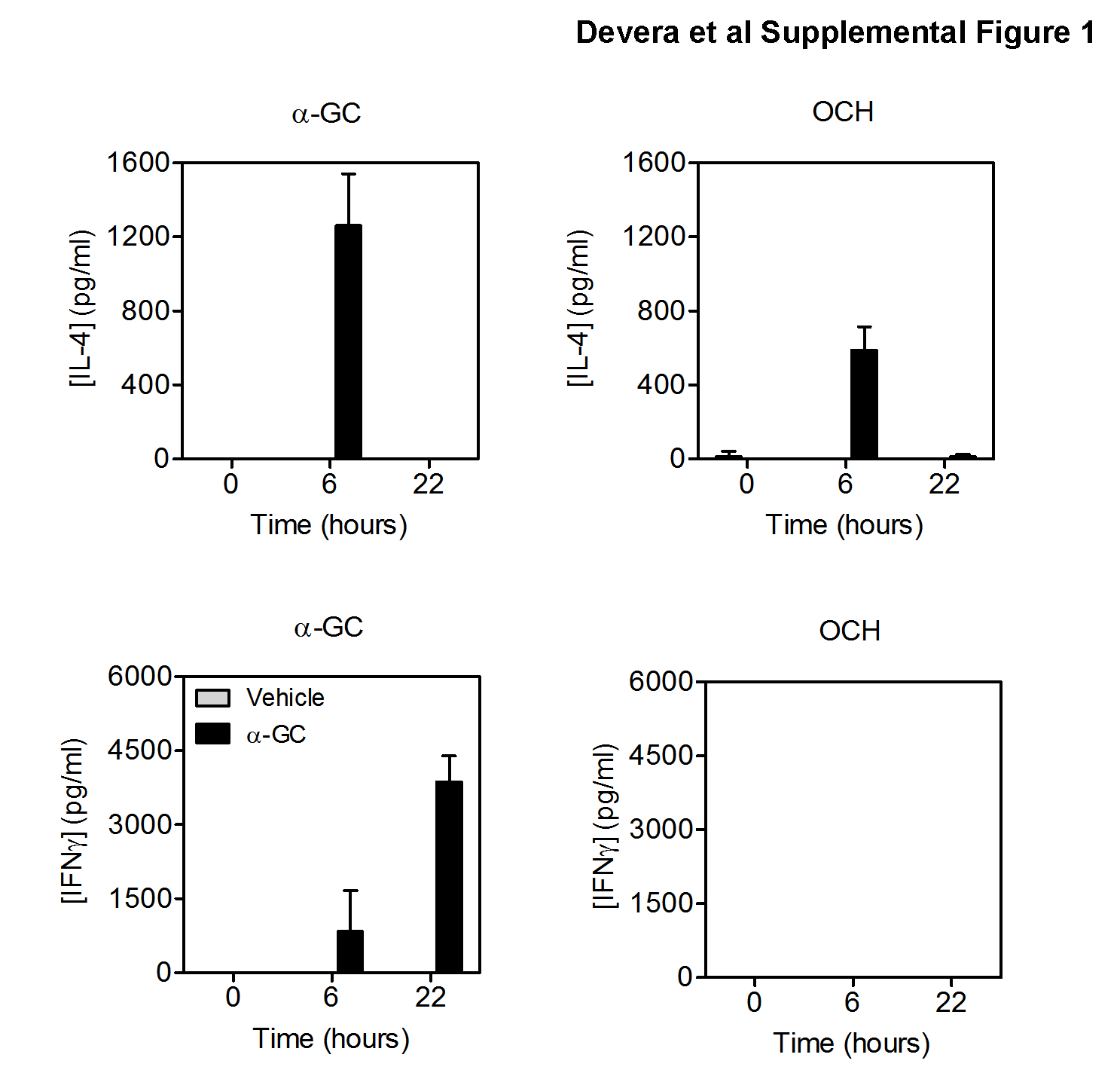

Supplement: Figure S1 — Polarized Th2 response in mice stimulated with OCH. C57Bl/6 mice were immunized i.p. with vehicle (PBS/Tween), vehicle plus α-GC, or vehicle plus OCH. Sera were collected prior to immunization and at 6 and 22 h thereafter. Concentration of IL-4 and IFNγ in the samples was then determined by sandwich ELISA. Data shows mean cytokine concentration for 3 (α-GC) and 5 (OCH) mice per group. CD1d−/− were also treated but did not elicit any measurable response to α-GC or OCH (not depicted). (TIF) [file pone.0023817.s001.tif]

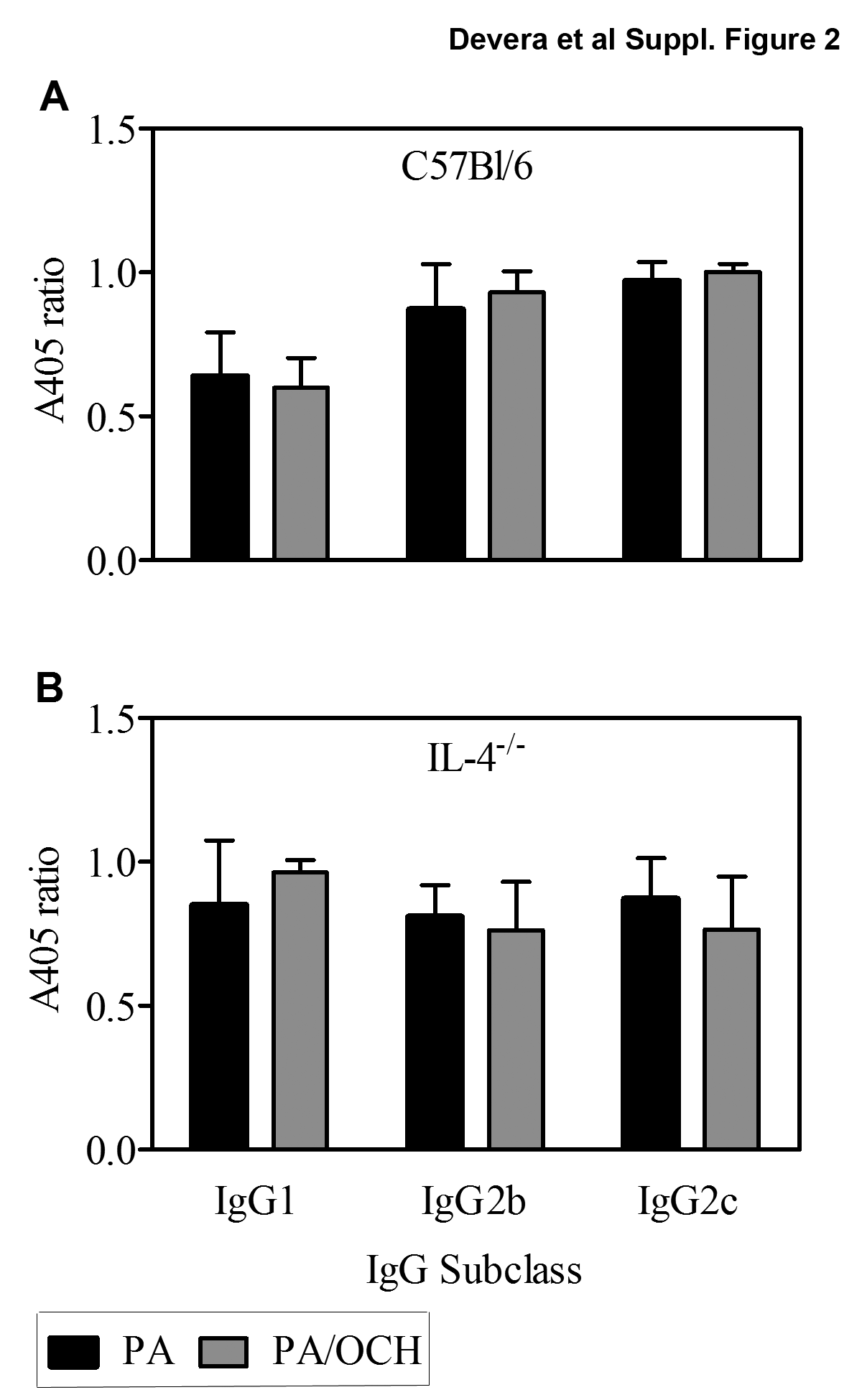

Supplement: Figure S2 — Affinity of PA-specific IgG1, IgG2b or IgG2c are not affected by OCH or by lack of IL-4. In vivo d 45 sera from experiment reported in Figure 4 were assessed by ELISA. Plates were coated with PA at a final concentration of 2 μg/ml and 20 μg/ml respectively. Sera at a 1/1000 dilution were then incubated on the plate with both PA concentrations before detection of Ab sub-classes. Bar graphs indicate the mean ±SD ratio of the A405 for samples applied to the 2 μg/ml wells versus the 20 μg/ml wells indicating the proportion of high affinity Ab in the samples. (TIF) [file pone.0023817.s002.tif]

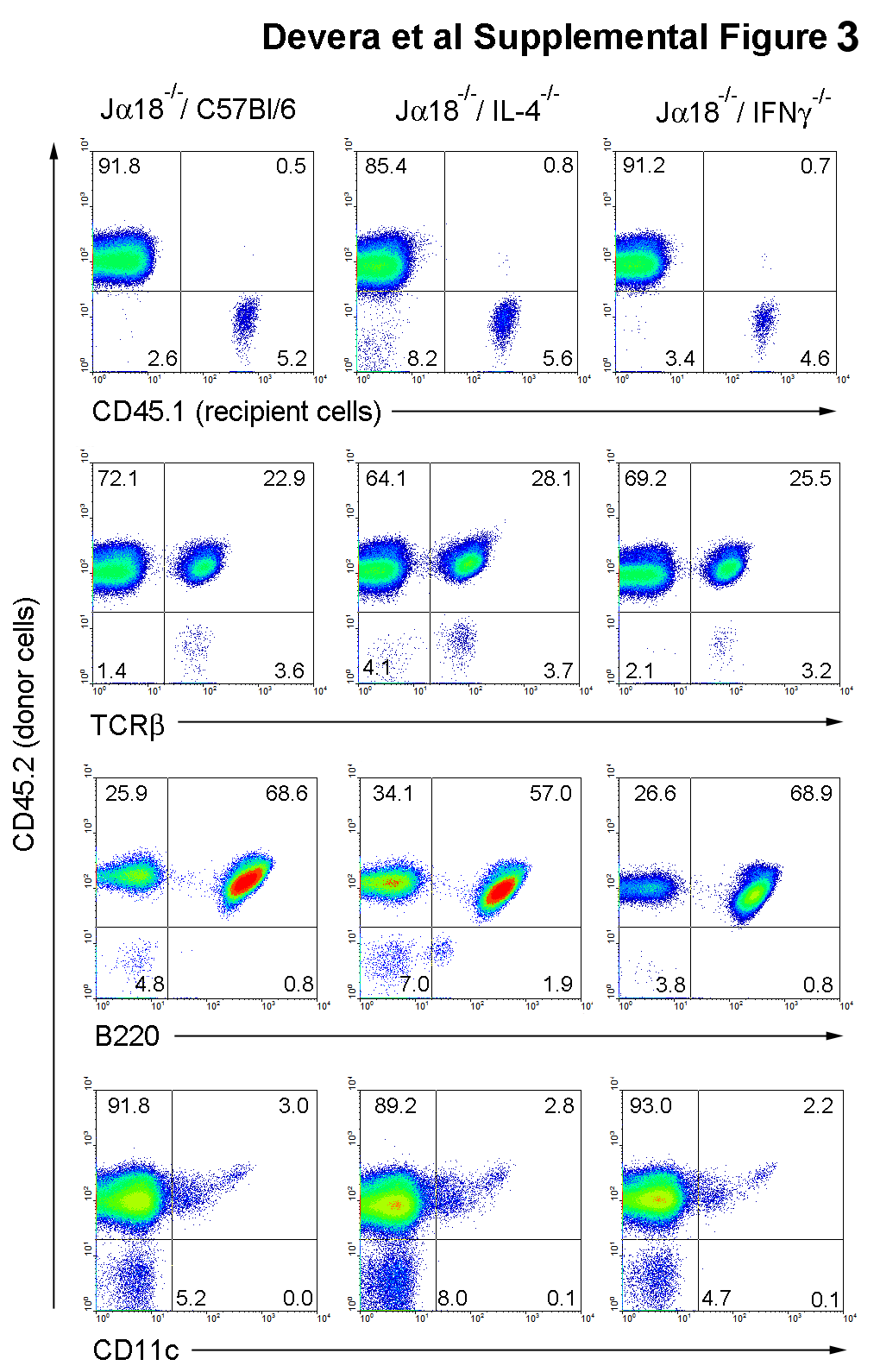

Supplement: Figure S3 — Effective reconstitution of the hematopoetic compartment following lethal irradiation. Spleens were obtained from immunized Jα18−/−/C57Bl/6, Jα18−/−/IL-4−/− and Jα18−/−/IFNγ−/− chimeric mice and analyzed by flow cytometry. Top row shows CD45.2+/+ donor cells and residual CD45.1+/+ recipient cells in re-constituted mice. Second row shows re-constitution with donor-derived T cells. Third row shows re-constitution with donor-derived B cells. Fourth row shows re-constitution with donor-derived dendritic cells. (TIF) [file pone.0023817.s003.tif]

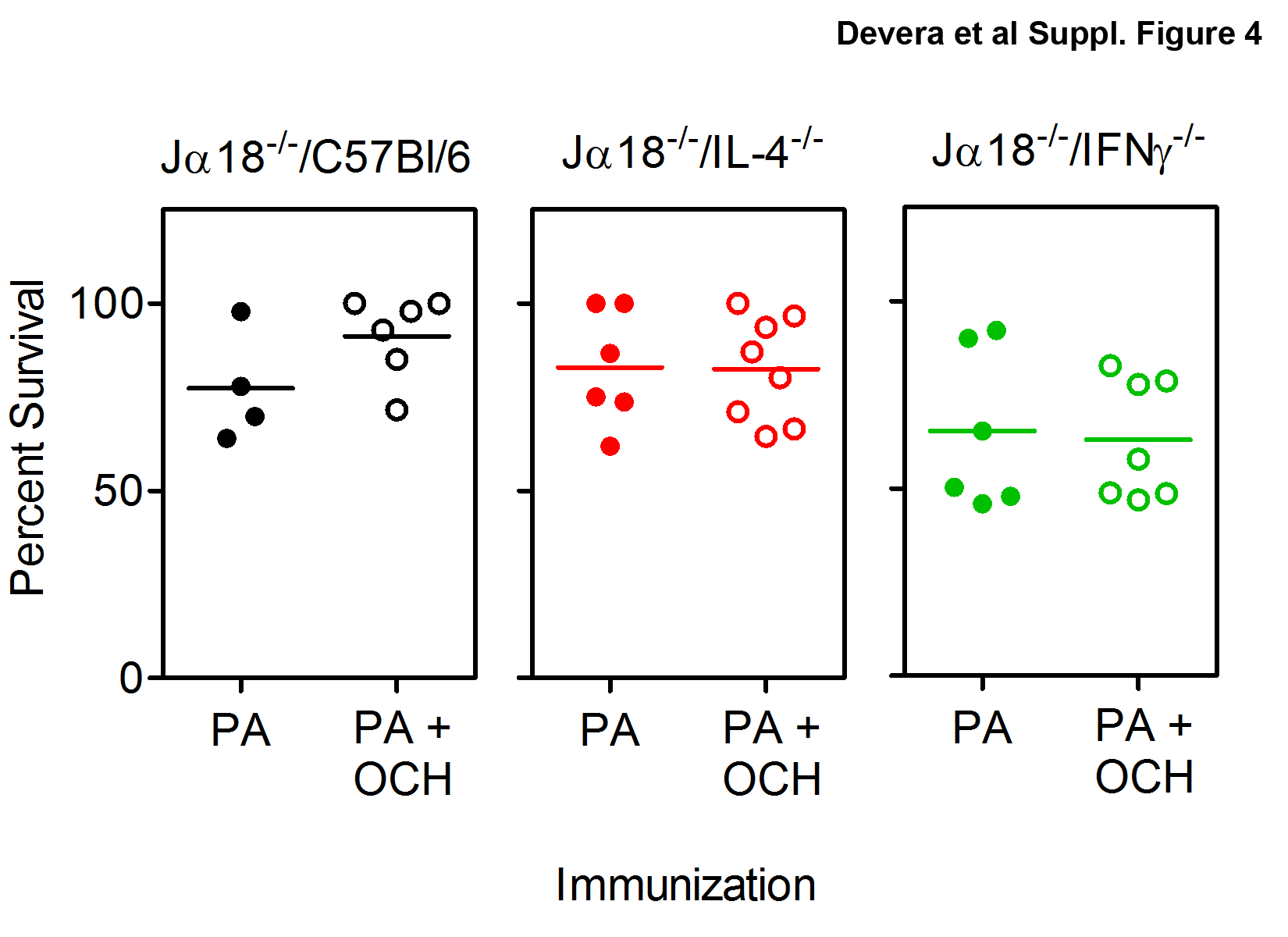

Supplement: Figure S4 — In vitro neutralization of lethal toxin by sera from PA-immunized chimeric mice. The d 45 sera from the experiment in Figure 6 were tested for their ability to protect RAW267.4 macrophages from LT toxicity. Graph shows neutralization by sera from Jα18−/−/C57Bl/6, Jα18−/−/IL-4−/− and Jα18−/−/IFNγ−/− chimeras. Each data point represents an individual mouse and the mean survival of the RAW267.4 macrophages following treatment with LT and sera is indicated. One of the sera from the Jα18−/−/C57Bl/6 group immunized with PA was not analyzed due to an insufficient amount of sample remaining. (TIF) [file pone.0023817.s004.tif]
